# Strong tribo-piezoelectric effect in bilayer indium nitride (InN)

**DOI:** 10.1038/s41598-021-98130-5

**Published:** 2021-09-21

**Authors:** Md. Sherajul Islam, Md. Yasir Zamil, Md. Rayid Hasan Mojumder, Catherine Stampfl, Jeongwon Park

**Affiliations:** 1 Department of Electrical and Electronic Engineering, Khulna University of Engineering and Technology, Khulna, 9203 Bangladesh; 2 Department of Materials Science and Engineering, Khulna University of Engineering and Technology, Khulna, 9203 Bangladesh; 3 School of Physics, The University of Sydney, Camperdown, NSW 2006 Australia; 4 Department of Electrical and Biomedical Engineering, University of Nevada, Reno, NV 89557 USA; 5 School of Electrical Engineering and Computer Science, University of Ottawa, Ottawa, ON K1N 6N5 Canada

**Keywords:** Energy science and technology, Materials science, Nanoscience and technology

## Abstract

The high electronegativity between the atoms of two-dimensional (2D) group-III nitrides makes them attractive to demonstrating a strong out-of-plane piezo-electricity effect. Energy harvesting devices can be predicted by cultivating such salient piezoelectric features. This work explores the tribo-piezoelectric properties of 2D-indium nitride (InN) as a promising candidate in nanogenerator applications by means of first-principles calculations. In-plane interlayer sliding between two InN monolayers leads to a noticeable rise of vertical piezoelectricity. The vertical resistance between the InN bilayer renders tribological energy by the sliding effect. During the vertical sliding, a shear strength of 6.6–9.7 GPa is observed between the monolayers. The structure can be used as a tribo-piezoelectric transducer to extract force and stress from the generated out-of-plane tribo-piezoelectric energy. The A–A stacking of the bilayer InN elucidates the highest out-of-plane piezoelectricity. Any decrease in the interlayer distance between the monolayers improves the out-of-plane polarization and thus, increases the inductive voltage generation. Vertical compression of bilayer InN produces an inductive voltage in the range of 0.146–0.196 V. Utilizing such a phenomenon, an InN-based bilayer compression-sliding nanogenerator is proposed, which can tune the generated tribo-piezoelectric energy by compressing the interlayer distance between the InN monolayers. The considered model can render a maximum output power density of ~ 73 mWcm^−2^ upon vertical sliding.

## Introduction

In recent years, nanogenerator and nanoscale energy harvesting devices are attracting tremendous attention due to their promising applications in low-power electronics and self-powered sensors^1–9^. In a nanogenerator, electrical energy can be attained from mechanical or thermal energy by small-scale physical changes. Two lucrative ways to form a nanogenerator are the consideration of the piezoelectric^8,10–12^ and triboelectric^8,13–16^ effects. When mechanical deformation is enforced on semiconductor materials, polarized charge is generated via the piezoelectric effect. In this context, vertical in-plane piezoelectricity in Janus transition metal dichalcogenides (TMDs)^15,17^ materials has been observed. Such a piezoelectricity phenomenon can be used to formulate new nanogenerators. Piezoelectrical nanogenerators have also been formulated utilizing interfacial ZnO nanostructures^11^, a lateral ZnO nanowire array^13^, and lead zirconate titanate nanofibers (PZT)^9^. In the case of the triboelectric effect, with the variation in the mechanical contact or friction between two layers of nanomaterials, temporary static charges are generated. Two triboelectrically charged surfaces can be considered to form a triboelectric nanogenerator^18–20^ utilizing in-plane sliding^21–23^ or vertical movement^24,25^ between them. The formulation of nanogenerator often involves both piezoelectric polarization and triboelectric charges. The tribo-piezoelectric phenomenon is demonstrated by Karumuthil et al., where they have taken the poly dimethyl siloxane-based hybrid nanocomposite^8^ and have reported enhanced current characteristics with improved mechanical to electrical energy conversion efficiency. Energy conversion efficacy can also be improved by incorporating an additional layer^21^ of nanomaterial between the electrodes and their substrates in a nanogenerator. New nanomaterials and structural patterns are considered to identify versatile and efficient tribo-piezo nanogenerators.

To develop nanoscale energy harvesting devices, two-dimensional (2D) materials^26–30^ are fascinating and promising due to their extraordinary features such as high electron mobility, large quantum spin hall effects, high carrier mobility, and very low thickness. In particular, transition metal dichalcogenides^31^ and Janus monolayer material WS-X (X = Se and Te)^32^ can demonstrate vertical and in-plane piezoelectrical properties. Among other 2D materials, group-III nitrides are prominent due to their diverse applications in the field of nanoelectronics, optoelectronics, spintronics, and heterojunction devices fabrications^28,29,33,34^. Since the group-III nitrides are electronegative in nature, it is predicted that these materials will demonstrate significant polarization with external mechanical disposition^35–40^. To this extent, Nan et al.^41^ have investigated piezoelectricity in 2D hexagonal boron nitride nanosheets (BNNS) and posited that piezoelectricity in BNNS strongly depends on the microscopic shape of the structure. In a single layer InN nanowire nanogenerator, a piezoelectric potential as high as 1 V can be obtained, and the polarity of the potential depends on the direction of the applied transverse force^42^. Guo et al.^43^ have demonstrated that the surface hydrogenated 2D group-III nitrides (AlN, GaN, InN) show both in-plane and out-of-plane piezoelectrical properties with external strain. Nevertheless, a detailed understanding of the contribution of both the triboelectric and piezoelectrical effects in the formation of nanogenerators of 2D group-III nitrides is still scarce.

Among the group-III nitrides, 2D-InN is lucrative due to its graphene-like hexagonal structure, strong quantum confinement, and stronger in-plane and out-of-plane excitonic effects with binding energies substantially greater than the room temperature thermal energy^44–47^. Moreover, InN can demonstrate optoelectrical properties around the visible to infrared spectral ranges^46^ and retains a 2D electron gas in the quantum well, a property well cherished for making high electron mobility transistors^48^. The large deviation between the electronegativity of In (1.78) and N (3.04) atoms predicts InN to display strong polarization in the milieu of in-plane external mechanical force and strain. However, to apply this material to nanogenerator applications, a detailed investigation of mechanical deformation and friction to generate electrical potential is an essential prerequisite.

In this work, the tribo-piezoelectricity effect of bilayer InN is explored through first-principles density functional theory investigations. At two separate interlayer distance points, a mutual transverse sliding effect is incorporated. It is observed that transverse sliding along a special direction can engender vertical polarization. Strong out-of-plane piezoelectricity can be achieved and tuned by changing the interlayer distance between the bilayer. With a decrease in the interlayer distance, the out-of-plane piezoelectric charge polarization increases and thus, renders the in-plane sliding energy barrier higher. The effect of the sliding variation results in charge transfer and charge redistribution between the layers. Moreover, the shear strength, the polarization deviation, and the energy variation with change in the interlayer distance demonstrate tunable tribo-piezoelectric properties. Since a deviation in the interlayer displacement can engender out-of-plane piezoelectricity, a compressive-sliding nanogenerator^17^ with an InN bilayer structure is proposed based on the tribo-piezoelectric effect and can render an electrical potential as high as ~ 0.2 V.

## Methods

A bilayer of InN is initially considered to investigate the mechanical deformation and friction. The rhombus unit cell of bilayer InN consists of 2-In and 2-N atoms. After structural optimization and relaxation, the bottom InN monolayer is kept fixed, and transverse sliding is incorporated into the top InN layer. As a result, the structure translates to different places in the XY-plane. The relaxation process is carried out for each position, and a constant interlayer spacing is maintained between N atoms in the top and bottom layers. During the in-plane sliding around the x–y axis of the top layer at an interlayer distance, the potential energy surface (PES) is calculated by:

1 $$E_{PES} \left( {x,y} \right) = E\left( {x,y} \right) - E_{lowest}$$

where $E_{PES} \left( {x,y} \right)$ represents the PES at the (*x, y*) coordinate. $E\left( {x,y} \right)$ refers to the energy obtained at the (x, y) coordinate and $E_{lowest}$ is the lowest energy of the system. The relative vertical polarization at a specific (x, y) coordinate can be quantified by considering the induced dipole moment as:

2 $$P_{d} = \frac{D}{A}$$

With D being the vertical dipole moment and A is the area of the unit cell. In the initial equilibrium state, because of the higher electronegativity of the N atoms, the charge shifts to the N atoms of the InN monolayers and causes out-of-plane electric charge polarization. Thus, the initial vertical polarization ($P_{Fn}$) of the InN bilayer can be set at 0. The polarization deviation surface (PDS) with in-plane sliding can then be calculated from the magnitude of vertical polarization by:

3 $$\Delta P\left( {x, y} \right) = P_{d} \left( {x, y} \right) - P_{Fn}$$

Here $\Delta P\left( {x,y} \right)$ and $P_{d} (x, y$) are the PDS and the vertical polarization at the (x, y) coordinate. When the sliding friction between the layers is incorporated, a higher vertical polarization is predicted. The amount of shear strength relative to the sliding of the layer can be obtained from the relation:4 Here  is the attained shear strength from the maximum amount of static resistance ($F_{mr}$), which acts on the InN monolayer within area $A$, during the sliding process.

All the calculations in this work are performed using density functional theory (DFT) employing the plane wave self-consistent field (PWscf) package of Quantum Espresso^49,50^. The Perdew-Burke-Ernzerhof (PBE)^51^ functional for the generalized gradient approximation (GGA) is used to describe the exchange–correlation functional. In the PWscf code, Kresse-Joubert projector augmented wave (KJPAW)^52^ potentials are used to describe the electron–ion interactions. The optB86b-vdW^53,54^ functional is employed to represent the Van-der Waals (vdW) weak intermolecular interaction. To ensure accurate simulation for the energies and interatomic distances at the equilibrium, the original PBE vdW-DF is modified by optB86b-vdW interaction^53–55^. The Brillouin zone integration is performed using a 2D-centered Monkhrost-Pack^56^ k-mesh grid of 15 × 15 × 1. For accurate convergence of the ground state electron density, an energy cut-off value of 10^−8^ a. u. is used. The kinetic energy cut-offs for the wave function and charge densities are set at 50 Ry and 400 Ry, respectively. Moreover, a force convergence threshold of 10^−3^ eV/nm is used for accurate stress and force calculations. A 15 Å vacuum region is used to ensure no direct interaction between the monolayers and bilayers.

## Results and discussion

**Figure 1:**
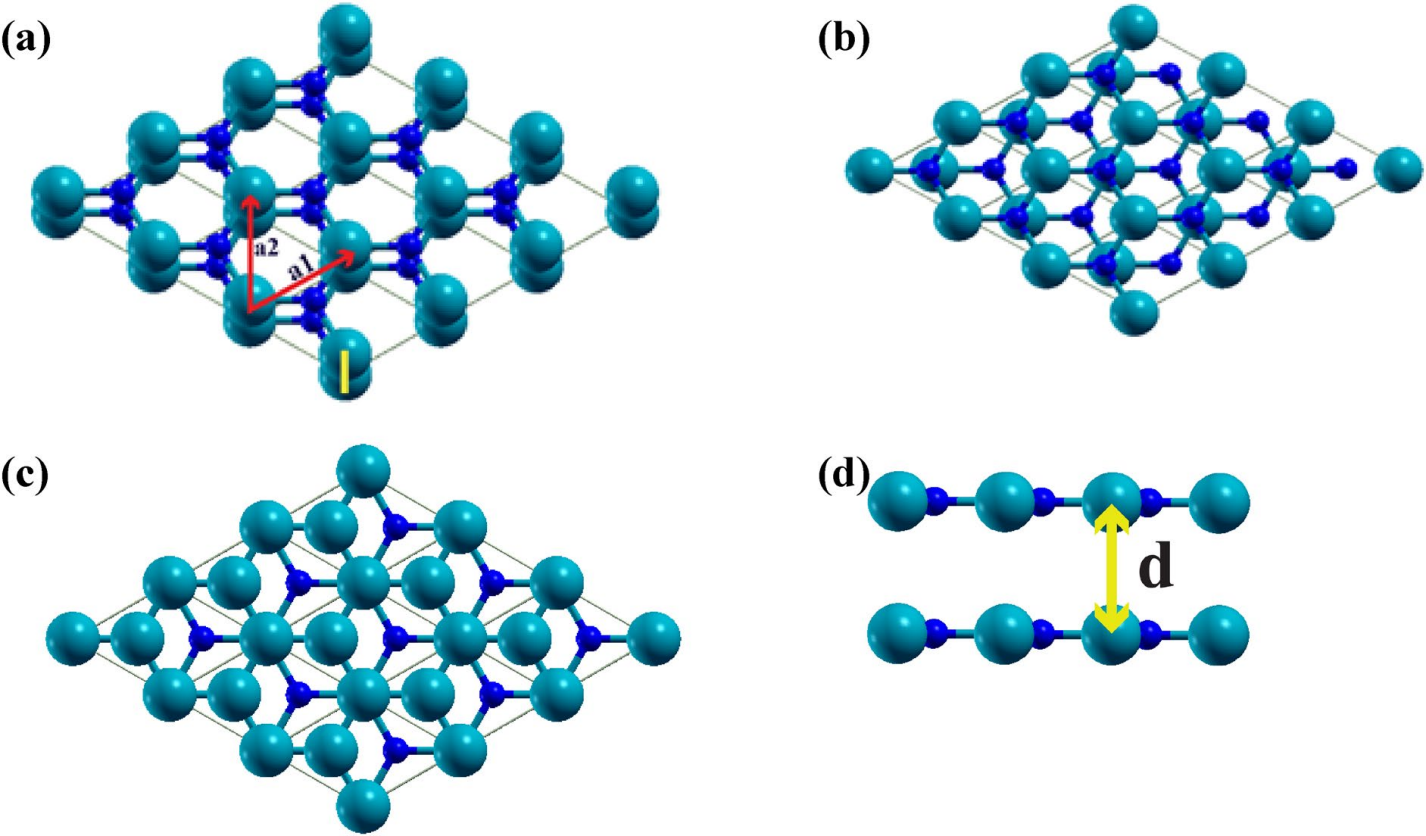
Top view of the (**a**) A–A, (**b**) A–B, and (**c**) A–C stacking patterns. (**d**) Side view of the bilayer showing the interlayer distance ‘d’ between the monolayers. Here, the blue and cyan colors refer to N and In atoms, respectively. a_1_ and a_2_ denote the in-plane lattice constants.

The investigation starts with the structural optimization of the InN monolayer. After relaxation, the obtained lattice constant is 3.58 Å with a zero buckling height. Our result complies well with the result of Sahin et al.^57^, where they obtained a lattice constant for InN of 3.57 Å with the local density approximation (LDA). Two energetically stable structures, A–B and A–C, are found for bilayer InN, which corresponds well with earlier study^46^. In the A–B stacking pattern, the N and In atoms are placed over the In atom and the center of the hexagon in the bottom layer, whereas, in the A–C stacking, the In and N atoms of the top layer are placed at the N atom and the center of the hexagon in the bottom layer, respectively, as shown in Fig. 1. The InN monolayers are separated by an interlayer distance measured between the top and bottom N atoms. The stable interlayer distance for the A–B and A–C patterns are found as 2.81 Å and 2.80 Å, respectively, by the calculation of the binding energy with respect to interlayer distance. Since the minimum binding energy point corresponds to the (local) equilibrium geometry, it will result in stable electronic properties^58–61^. Besides, the calculation of the binding energy of a material structure provides significant information about the stability of the material. A negative binding energy is obtained by a stable interlayer distance, with a higher negative value implying more stability than a smaller value. For the InN bilayer structure, we obtained negative (stable) binding energies when the interlayer distance was in the range of 2.41 to 2.81 Å. This range of interlayer distance is then considered as a criterion for selecting the range for the stable interlayer distance to employ vertical compressive sliding of the top InN layer to attain higher maximum energy corrugation.

**Figure 2:**
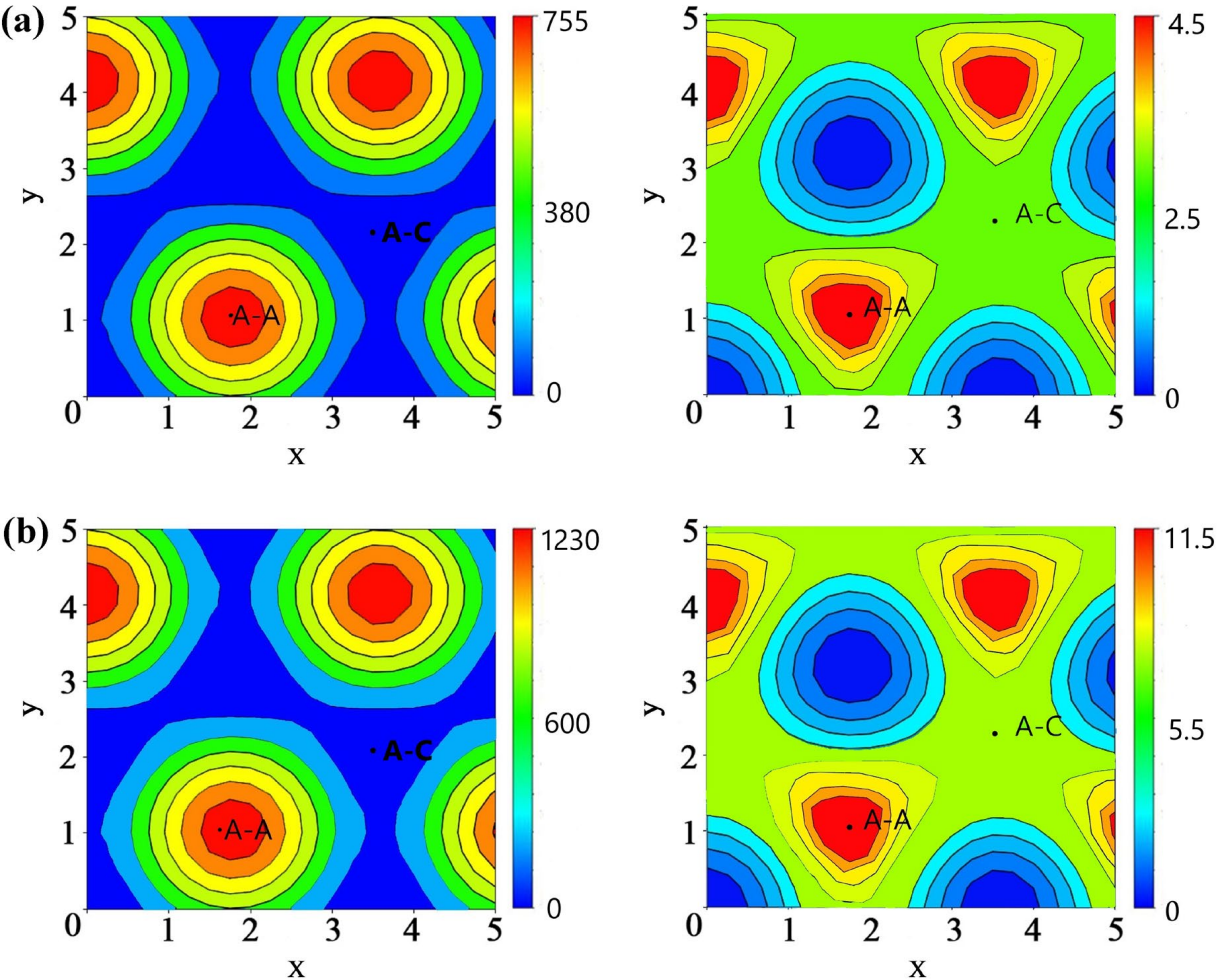
The potential energy surface (PES) (on-left) and polarization deviation surface (PDS) (on-right) for in-plane sliding of the InN bilayer when the interlayer distance is (**a**) 2.81 Å and (**b**) 2.51 Å.

The effect of transverse sliding is investigated at the two different interlayer distances (2.81 Å and 2.51 Å). The potential energy surface (PES) and polarization deviation surface (PDS) at various sliding locations are calculated and depicted in Fig. 2. The PES and PDS plots are periodically repeating in nature, which commensurates with the equilibrium geometry of the flat honeycomb structured group-III 2D nitrides^37^. According to Fig. 2, whenever the initial pattern slides to the resemblance of the A–A stacking pattern, very high vertical polarization is demonstrated. Moreover, such high vertical polarization increases for a low interlayer distance. On the other hand, the A–B stacking pattern renders very low vertical polarization with a change in the interlayer distance. Moreover, the A–B stacking has a substantially weaker response to interlayer distance than the A–C stacking. Thus, the A–A stacking pattern will attain the maximum polarization enhancement, and the A–B stacking pattern is taken as the initial state. Nonetheless, without external mechanical stimulus or restraint, the A–A stacking is not energetically favorable. During the in-plane sliding of the A–B pattern, only a lateral driving force that is higher than the interlayer shear strength and resistive force can trigger a transition to the A–A pattern. Interlayer sliding and accompanying resistance are tribological phenomena, whereas shifting to A–A stacking with a fixed interlayer distance enhances vertical polarization significantly. As a result, the out-of-plane polarization amplification caused by in-plane interlayer sliding in the InN bilayer can be regarded as a tribo-piezoelectric phenomenon. The piezoelectricity is the result of tribological energy conversion, which overcomes the energy barrier of sliding resistance. To drive the InN bilayer to the A–A state, which can be seen of as the conversion of tribological energy to electricity, external driving force and pressure are required.

At a fixed interlayer distance, the process of overcoming the energy barrier related to resistive force and interlayer shear strength maintains the triboelectric effect. As such, the vertical plane sliding that generated out-of-plane vertical polarization will be the result of the tribo-piezoelectric effect. Unlike previous studies^8,11^, where the triboelectric effect renders static charges, in our study, the tribological energy engenders piezoelectricity. Similar tribo-piezoelectric behavior has been observed in the Janus transition metal dichalcogenide bilayers by Cai et al.^17^. The energy corrugation (∆E) in the PES of the InN bilayer thus is a function of vertical interlayer resistive frictions; a higher interlayer resistance and friction will generate a larger energy corrugation^36^.

**Figure 3:**
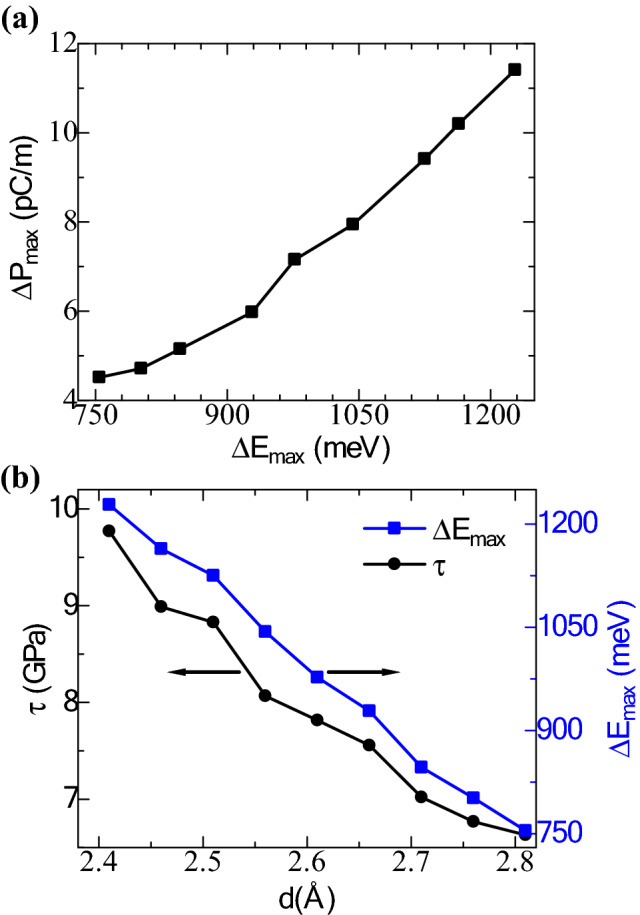
(**a**) The maximum polarization changes (pC/m) with the maximum sliding energy barriers (meV). (**b**) The variation of the interlayer shear strength (black line with filled circles) and maximum sliding energy barriers (blue line with blocks) with change in the interlayer distance of the InN bilayer.

The amount of tribo-piezoelectricity generation depends on the energy corrugation (∆E). Figure 3a represents the maximum polarization deviation (∆P_max_) with maximum energy corrugation (∆E_max_) with the compressive variation of the interlayer distance from 2.81 Å to 2.41 Å. According to Fig. 3a, the maximum polarization deviation increases linearly with an increase in the maximum energy corrugation. The higher the energy required to overcome the interlayer sliding friction, the larger value of vertical polarization will be achieved. The maximum static resistance force acting on the sliding process can be utilized to calculate the relative shear strength from Eq. (4). Figure 3b is a depiction of the shear strength related to an interlayer distance within the range of 2.41 Å to 2.81 Å. The maximum energy barrier deviation (754–1227 meV) and shear strength (6.6–9.7 GPa) during the vertical compressive sliding of the InN layer are higher than those obtained with the Janus transition metal dichalcogenide (TMD) bilayers ((180–480 meV) and (2.5–7.2 GPa), respectively^17^. A higher vertical polarization value (4.5–11.4 pC/m) can also be obtained with the InN bilayer than the Janus TMD bilayers (0.6–2.3 pC/m)^17^. A reduction in the interlayer distance renders a quadratic increase in both shear strength and maximum corrugated energy values. Thus, vertical compressive sliding is an efficient way to obtain higher triboelectric to electrical energy conversion. However, a structural change has been noticed in this process due to the effect of in-plane strain caused by vertical stress. The change in interlayer distance alters the interaction among the atoms of the bilayer; influence becomes stronger when the bilayer is placed at a lower interlayer spacing than the optimized value. When the interlayer distance was at its optimized point (2.81 Å), the lattice constant was 3.587 Å. But decreasing the interlayer distance results in a reduction of lattice constant. At a spacing of 2.51 Å and 2.41 Å, the lattice constant reduces to 3.553 Å and 3.549 Å, respectively. The system is relaxed for each time upon application of vertical stress, and the changes in the lattice constant are taken into consideration in the calculations.

**Figure 4:**
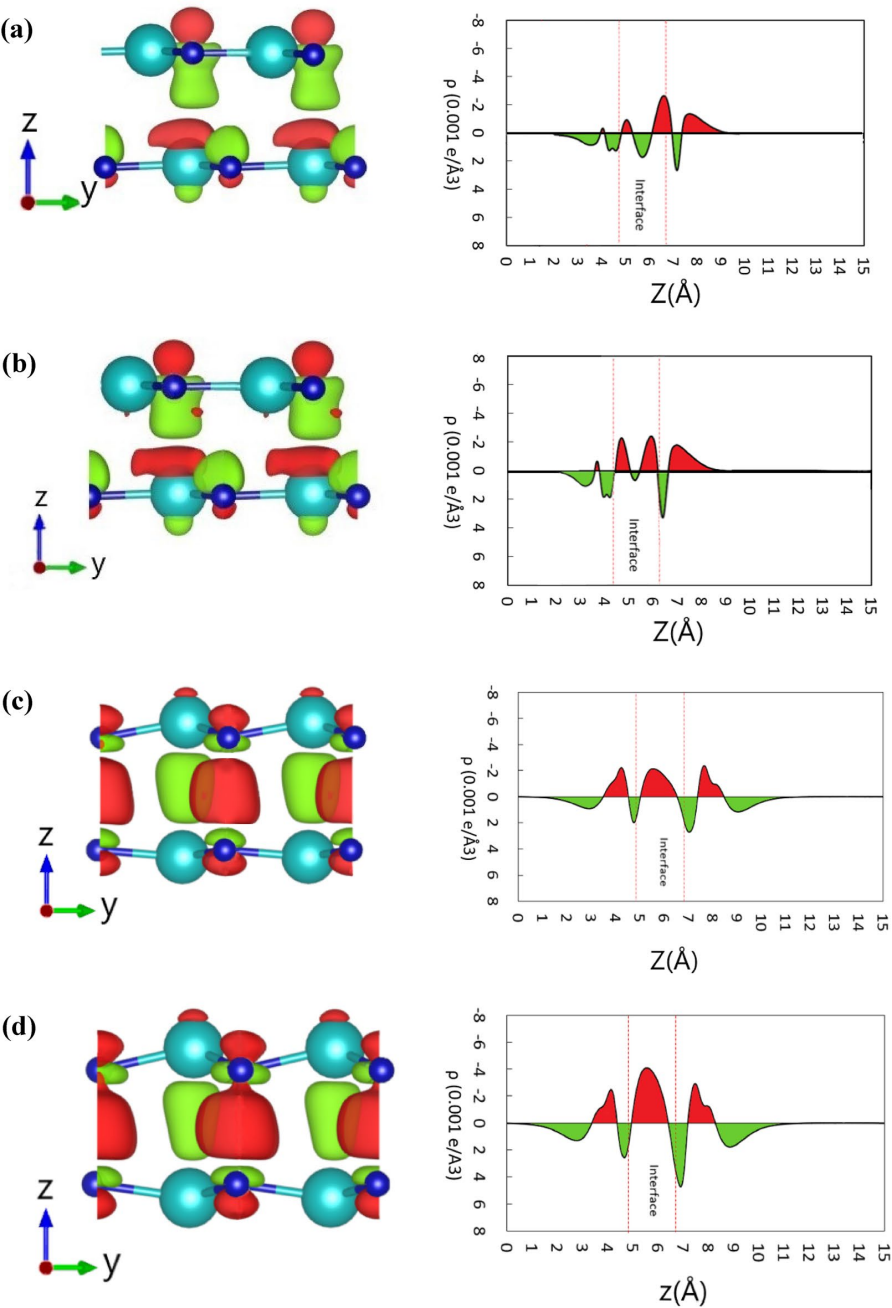
The charge density differences between the InN monolayers at 0.001 e/Å^3^. Here the upper two plots (**a**, **b**) refer to the A–B stacking pattern at an interlayer distance of 2.81 Å and 2.51 Å, respectively, and the lower two plots (**c**, **d**) refer to the A–A stacking pattern at an interlayer distance of 2.81 Å and 2.51 Å, respectively. Here, the red color refers to the charge accumulation, and the green color refers to the charge depletion.

To demonstrate the charge contribution to the observed tribo-piezoelectric property in the InN bilayer, the charge density difference (CDD) is investigated. Figure 4 represents the CDD between the InN monolayers at two interlayer distances (2.81 Å and 2.51 Å). The CDD is enumerated as $\Delta \uprho = \uprho _{{{\text{total }}}} - \rho _{{{\text{top}}}} - \uprho _{{{\text{bottom}}}}$, where $\uprho _{{{\text{total}}}}$ is the total charge density of the InN bilayer, $\uprho _{{{\text{top}}}}$ and $\uprho _{{{\text{bottom}}}}$ refers to the charge density associated with the top and bottom InN layers. It can be seen that, for both interlayer displacements, the charge accumulates near the N-atoms of the monolayers and at the surface between them. Moreover, when the A–B (Fig. 4a,b) stacking pattern slides into the A–A (Fig. 4c,d) stacking pattern, the magnitude of the charge accumulation around the N-atoms drastically increases. For the A–A stacking pattern, with a lower interlayer distance (2.51 Å), the CDD can be improved significantly from that obtained at an interlayer distance of 2.81 Å. A large amount of charge accumulation around the N-atoms demonstrates higher out-of-plane polarization of the A–A stacking pattern.

**Figure 5:**
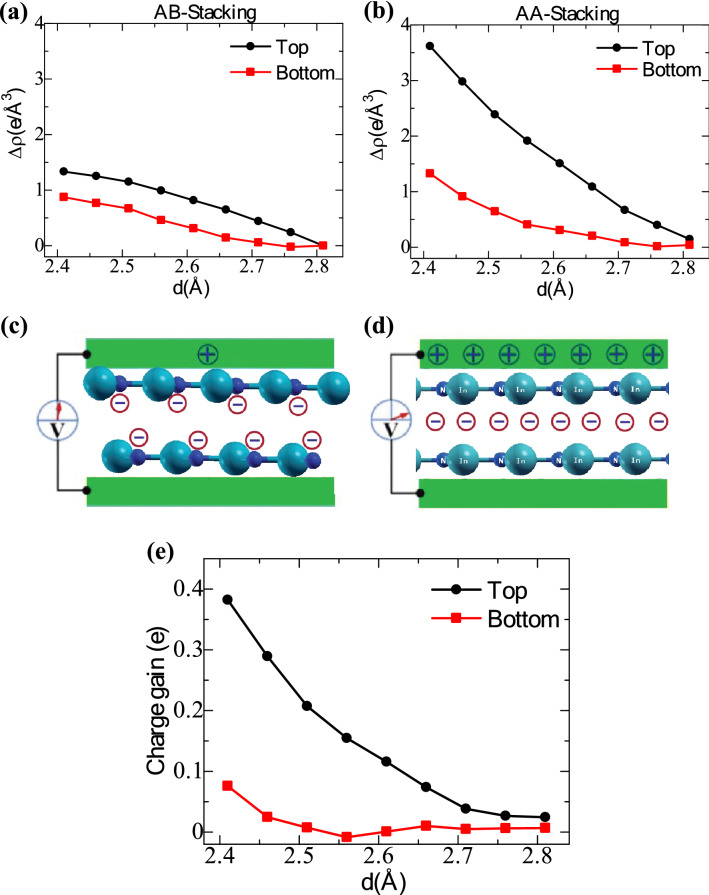
(**a**, **b**) Average charge density differences at 0.001 e/Å^3^ on the two monolayers of the InN bilayer for (**a**) A–B stacking and (**b**) A–A stacking with the change in the interlayer distance. Relative inductive charge and voltage that are accumulated at the electrodes that contact with the surface of the top and bottom layers of the (**c**) A–B stacking and (**d**) A–A stacking. (**e**) Charge gain during the sliding of the top and bottom layer of the InN bilayer.

With the variation of the interlayer distance, the average charge density differences for the top and bottom monolayers of InN are also observed. For this process, initially, zero normal force (F_n_ = 0) is applied in the Z-direction. The charge density differences within the monolayers can be defined as:

5 $$\Delta {\uprho }^{{\text{T}}} = \left( {{\uprho }_{{\text{N}}}^{{\text{T}}} - {\uprho }_{{\text{N}}}^{{T, F_{n} = 0}} } \right)$$

6 $$\Delta {\uprho }^{{\text{B}}} = \left( {{\uprho }_{{\text{N}}}^{{\text{B}}} - {\uprho }_{{\text{N}}}^{{B, F_{n} = 0}} } \right)$$

where $\Delta {\uprho }^{{\text{T}}}$ and $\Delta {\uprho }^{{\text{B}}}$ are the CDD at the top and bottom InN monolayers, ${\uprho }_{{\text{N}}}^{{\text{T}}}$ and ${\uprho }_{{\text{N}}}^{{\text{B}}}$ are the average charge densities of the N-atoms at the Z-direction with the interlayer variation, and ${\uprho }_{{\text{N}}}^{{{\text{T}}, F_{n} = 0}}$ and ${\uprho }_{{\text{N}}}^{{{\text{B}}, F_{n} = 0}}$ with zero normal forces. Figure 5a,b represent the average charge density variations with interlayer distances in the range of 2.81–2.41 Å. It can be seen that a lower interlayer separation causes a higher average charge density within the layers. For the A–B stacking pattern, with compression of the vertical displacement between the layers, a higher amount of charge density is observed at the top layer than the bottom layer. At the same time, the charge density of the top layer dominates in the A–A stacking pattern. The charge distribution between the top and bottom monolayers of InN complies well with the CDD plot (Fig. 4). The charge redistribution between the layers and atoms can be considered for developing a compressive-sliding nanogenerator. Since the majority of the charge accumulates near the N-atoms, the electron density around the N-surface will be higher than the interface electron density between the layers. Attaching electrodes at the top and bottom surfaces can engender a small voltage, as shown in Fig. 5c. For the A–A stacking pattern, a higher accumulation of inductive charges appears near the interface, as shown in Fig. 5d. Thus, a larger voltage can be extracted by forming a nanogenerator with the A–A stacking pattern. Additionally, the charge gain at the top and bottom layers with the vertical sliding process is shown in Fig. 5e. The charge gain is calculated by subtracting the monolayer charges between the unit cell of the A–A and A–B stacking patterns.

**Figure 6:**
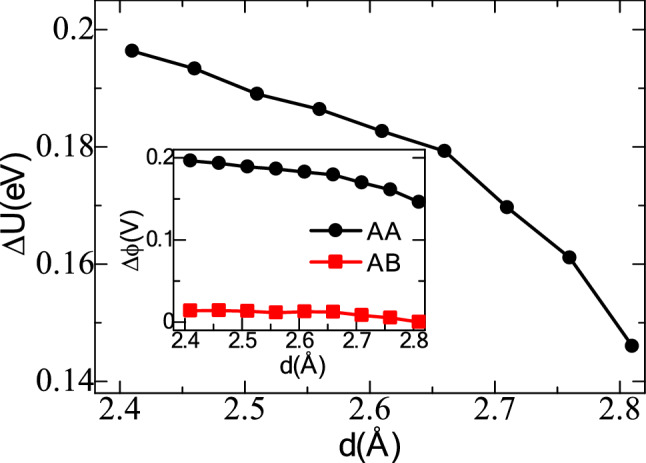
Variation of the electrostatic potential differences between the N-atoms of the top and bottom layers for AA stacking pattern. Inset shows the comparison of estimated electric voltages between A–B and A–A stacking patterns with compression of the interlayer distance of the InN bilayer from 2.81 to 2.41 Å.

Next, considering the A–A stacking pattern, the maximum generated voltage is enumerated by considering the electrostatic potential differences (∆U) between the N-atoms of the top and bottom layers. The change in the electrostatic potential differences with interlayer variation is found at the monolayer surfaces by:

7 $$\Delta {\text{U}} = \Delta {\text{U}}^{{\text{T}}} - \Delta {\text{U}}^{{\text{B}}}$$

where

8 $$\Delta {\text{U}}^{{\text{T}}} = \left( {{\text{U}}_{{\text{N}}}^{{\text{T}}} - {\text{U}}_{{\text{N}}}^{{T, F_{n} = 0}} } \right)$$

9 $$\Delta {\text{U}}^{{\text{B}}} = \left( {{\text{U}}_{{\text{N}}}^{{\text{B}}} - {\text{U}}_{{\text{N}}}^{{B, \; F_{n} = 0}} } \right)$$

where $\Delta {\text{U}}^{{\text{T}}}$ and $\Delta {\text{U}}^{{\text{B}}}$ are the electrostatic potential variation at the top and bottom InN monolayers, ${\text{U}}_{{\text{N}}}^{{\text{T}}}$ and ${\text{U}}_{{\text{N}}}^{{\text{B}}}$ are the electrostatic potentials around the N-atoms at the top and bottom surfaces of the monolayers and ${\text{U}}_{{\text{N}}}^{{{\text{T}}, F_{n} = 0}}$ and ${\text{U}}_{{\text{N}}}^{{{\text{B}}, F_{n} = 0}}$ are the corresponding cases at zero normal forces. According to Fig. 6, a decrease in the interlayer distance causes a higher vertical polarization and renders a higher electrostatic potential at the top and bottom layers. Throughout the process, an induced voltage ranging from 0.146 to 0.196 V can be obtained by considering the electrostatic potential and unit charges as φ = (∆U/q), where q is the unit charge, and φ is the induced voltage.

**Figure 7:**
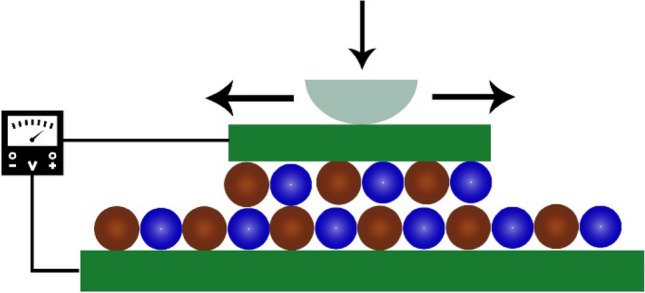
Schematic diagram of an InN-based compression-sliding motion nanogenerator model. The probe and electrodes are denoted by light cyan and green colors, respectively.

Finally, to apply the proposed bilayer InN structure as a nanogenerator, a compression-sliding motion model is constructed in Fig. 7. To experimentally develop the proposed nanogenerator, the nanoscale probing approach^23,62–64^ can be used to incorporate rotation or force on a monolayer, or a few layers of material, at a specific path and direction. Here a small InN flake slides on a large InN substrate according to the applied vertical and transverse motion via the probe tip. The bilayer InN is attached to two electrodes at two ends, and the top electrode is attached to a probe for motion control of the flake. The probe is configured as a conductor connecting the bottom electrode^65^. The commonly used Au-electrodes can be utilized in this model. Our further investigations reveal that the adhesion force between the Au electrode and the InN monolayer surface is much stronger than the force obtained between two InN monolayers, and the Au-contact has very little effect on the tribo-piezoelectricity of the InN bilayer (see Figs. S1 and S2 in Supplementary materials).

Our analysis and earlier studies on Au/2D-materials contact justify that the compression-sliding technique to be stable and resilient with the InN bilayer. The presence of the interlayer resistive force and sliding force converts mechanical energy into electrical energy. Thus, during the sliding process, tribological energy (E_tribo_) will contribute to the majority of the electrical energy (E_elec_) production. If a conversion co-efficient (δ) is taken, then the amount of E_elec_ production can be found from the relation,

10 $$E_{elec} = \delta E_{tribo} \approx Pt$$

where P refers to the amount of electrical power generated from the nanogenerator at a certain time (t). Since the sliding force is provided to the model in a specific direction, the schematic configuration of Fig. 7 can further be investigated as a spring-force model^66^. If ∆E_max_ is the maximum sliding force barrier for one sliding cycle, then the amount of triboelectric energy and output electrical power per sliding cycle can be found by^17^,

11 $$E_{tribo} = \frac{{x\Pi \Delta E_{max} }}{2a}$$

12 $$P \approx \frac{{E_{tribo} }}{t} = \frac{{x\Pi \Delta E_{max} }}{2at}$$

where *x* and *a* are the sliding displacements per cycle and lattice constant, respectively. The power generated from the InN nanogenerator thus depends on the sliding velocity (x/t) and the height of the maximum sliding force barrier (∆E_max_). When a higher sliding pressure is given to the model via the probe, the interlayer distance will decrease, and a higher output power will be obtained. In practice, the sliding velocity with the probe tips can range from 1 to 100 nm/s^17^. The power density coming from tribo-piezoelectricity can range from 738.22 to 73,822 μWcm^−2^ for the sliding velocity from 1 to 100 nm/s and the ∆E_max_ for the circumstance when the interlayer spacing is reduced to 2.51 Å, assuming that all the tribological energy converts to electrical energy. According to experimental observation^11^, a tribo-piezoelectric generator can provide a power density of 7 µWcm^-2^. Previous investigations have outlined the power density output from piezoelectric nanogenerators^66^ and triboelectric nanogenerators^67^ to be within the range of 4.41 to 5.92 μWcm^−2^ and 400 to 50,000 μWcm^−2^, respectively. The predicted InN bilayer nanogenerator shows a decent maximum power density and a considerable induced electrical output voltage, which is comparable to the theoretically and experimentally addressed triboelectric nanogenerators of Janus TMD bilayers^17^, GaN nanowire array^68^, *h*-BN polypropylene^69^, monolayer MoS_2_ flake^70^, and bilayer PVDF-TrFE and graphene oxide^66^, as outlined in Table 1.

**Table 1:** Comparison of the maximum power density and induced voltage obtained from the proposed tribo-piezoelectric nanogenerator structure with previously investigated structures.

Proposed material	Tribo-piezoelectric maximum power density (mWcm^−2^)		Peak induced output voltage (V)	
	Theoretical	Experimental	Theoretical	Experimental
Proposed InN bilayer	~ 73	–	0.196	–
Janus TMD bilayers^49^	~ 29.64	–	0.25	–
Monolayer MoS_2_ flake^70^	–	~ 2 × 10^–4^	–	0.015
GaN nanowire array^68^	–	–	–	0.15–0.35
h-BN polypropylene^69^	–	~ 29 × 10^–4^	–	2.3
Bilayer PVDF-TrFE and graphene oxide^66^	–	4.41 × 10^–3^	–	4

**Figure 8:**
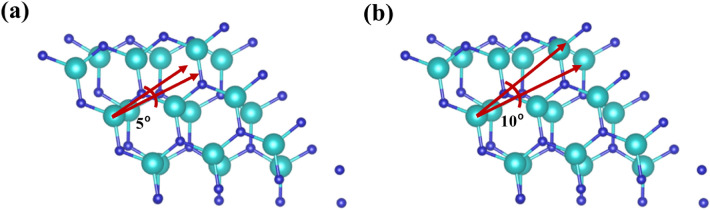
InN bilayer with upper layer is twisted at an angle of (**a**) 5° and (**b**) 10°. Here, the gray and cyan colors refer to N and In atoms, respectively.

Besides, we also extended our analysis by considering a 3 × 3 InN supercell bilayer structure that results in a power density of ~ 747.03 to 74,703 μWcm^-2^, which is slightly greater than the findings obtained with the InN unit cells. The induced voltage and polarization for the supercell structure are 0.148 V and 4.40 pC/m, respectively, whereas the values for the unit cell structure are 0.146 V and 4.38 pC/m. Thus, with increased surface area (large supercell structure), charge deviation increases, resulting in slightly higher induced voltage and maximum power density. Furthermore, a 10º and 5º twist on the upper InN layer has also been considered (Fig. 8). The maximum power density reaches to 713.58 μWcm-2 and 665.11 for the twist angles of 5º and 10º, respectively. The induced output voltage observed at 5º and 10º twists are ~ 0.108 and 0.100 V, respectively. Thus, a higher twist angle causes a reduction of induced voltage and maximum power density. As the twist angle increases (0º, 5º, and 10º), the polarization decreases (0.409, 0.408, and 0.404 pC/m, respectively), leading to the reduced induced voltage and power density.

The considered InN nanogenerator is a promising candidate in energy harvesting applications, especially for low-power electronics and self-powered sensors. In recent times, there have been tremendous improvements and investigations to fabricate InN based devices^71^, for instance, thin-film transistors^72^, lasers^73^, photovoltaic converters^74^, photodetectors^75^, and a number of terahertz-range devices^76^. Moreover, InN based nanowire^77^ and other structures^78,79^ are also getting improved day by day. Thus, the experimental fabrication of a 2D InN based tribo-piezoelectric nanogenerator is also expected to be feasible and sustainable in the near future.

## Conclusions

In summary, the bilayer 2D InN structure is investigated to demonstrate the tribo-piezoelectric effect through first-principles calculations. For a fixed interlayer distance, in-plane sliding of the top InN layer results in a large out-of-plane vertical polarization. Instead of generating static charges, the tribological energy engenders piezoelectric polarization. The A–A stacking patterns result in a higher tribo-piezoelectric energy than the A–B stacking pattern. The amount of tribological energy depends on the magnitude of shear strength and interlayer sliding resistive force. The obtained induced voltage can be as high as ~ 0.2 V during vertical compressive sliding of the top plane. Moreover, a compressive-sliding motion nanogenerator structure is proposed. The nanogenerator can result in a tunable electrical output of 738.22 to 73,822 μWcm^−2^ by varying the pressure on its probe tip. These investigations strongly demonstrate that the InN bilayer could be a stable and highly efficient nanogenerator for energy conversion and energy harvesting.

## Supplementary Information

Supplementary Information.
