# Supplementary material for: Strong tribo-piezoelectric effect in bilayer indium nitride (InN)
Source: Sci Rep. 2021 Sep 21;11:18669. doi: 10.1038/s41598-021-98130-5 (PMC8455586; doi:10.1038/s41598-021-98130-5)
Supplement: Supplementary file 1 — Supplementary Information. [file 41598_2021_98130_MOESM1_ESM.docx]

**Supplementary Materials**

**Strong tribo-piezoelectric effect in bilayer indium nitride (InN)**

Md. Sherajul Islam*^,a, d^, Md. Yasir Zamil^b^, Md. Rayid Hasan Mojumder ^a^, Catherine Stampfl^c^, Jeongwon Park^d,e^

^a^Department of Electrical and Electronic Engineering, Khulna University of Engineering &Technology, Khulna 9203, Bangladesh

^b^Department of Materials Science and Engineering, Khulna University of Engineering &Technology, Khulna 9203, Bangladesh

^c^School of Physics, The University of Sydney, New South Wales 2006, Australia

^d^Department of Electrical and Biomedical Engineering, University of Nevada, Reno, NV 89557, USA

^e^School of Electrical Engineering and Computer Science, University of Ottawa, Ottawa, ON K1N 6N5, Canada

***Corresponding author. Email:** [**sheraj_kuet@eee.kuet.ac.bd**](mailto:sheraj_kuet@eee.kuet.ac.bd) **(Md. Sherajul Islam)**

The effect of placing a metallic Au electrode on the InN monolayer is studied by considering a relaxed Au-supported InN structure, as shown in **Fig. S1**. The calculations are carried out in the same way as in the manuscript. We compressed the lattice constant of InN by ~ 4.7% to match the lattice constant of the Au substrate. The optimized interlayer spacing obtained between the Au and InN layer is 2.209 Å. Thus, the Au-InN interface will show physical adsorption. The calculated binding energies per In atom for the Au/InN and InN bilayer are -0.202 eV and -0.116 eV, respectively. The adhesion force between the Au electrode and the InN monolayer surface is much stronger than the force obtained between two InN monolayers. A similar result was also found the the Au-TMD interface^1^.

**
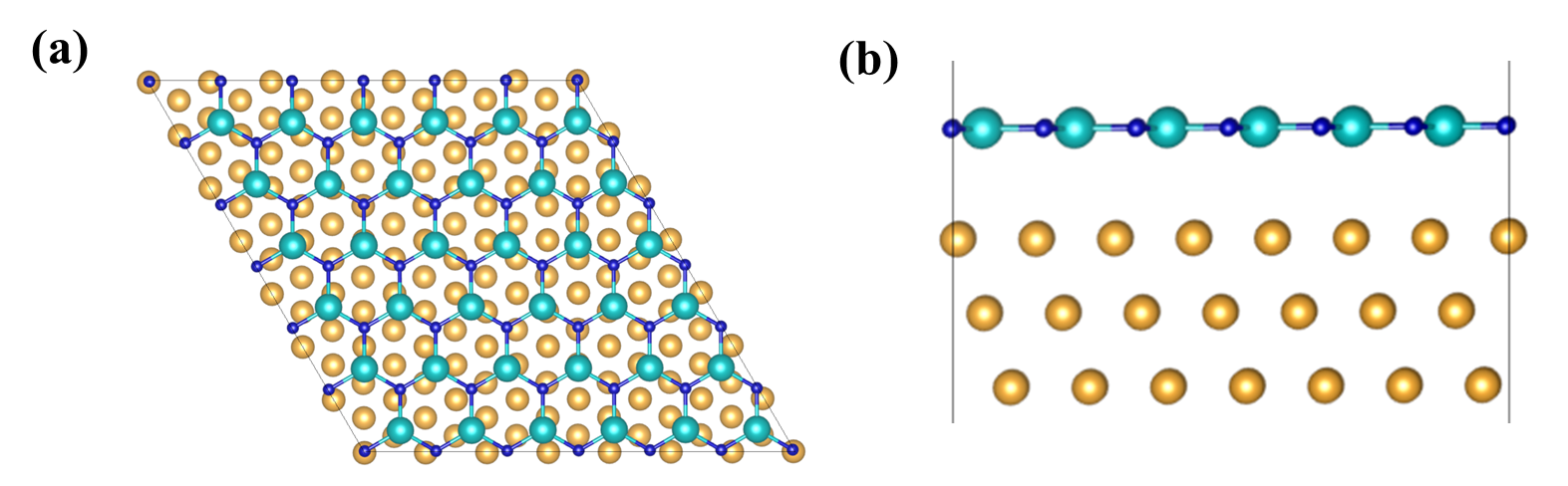
**

**Fig. S1.** Atomic configuration of Au-supported monolayer InN, (a) top view and (b) side view. Here, the blue, cyan, and golden colors refer to N, In, and Au atoms, respectively.


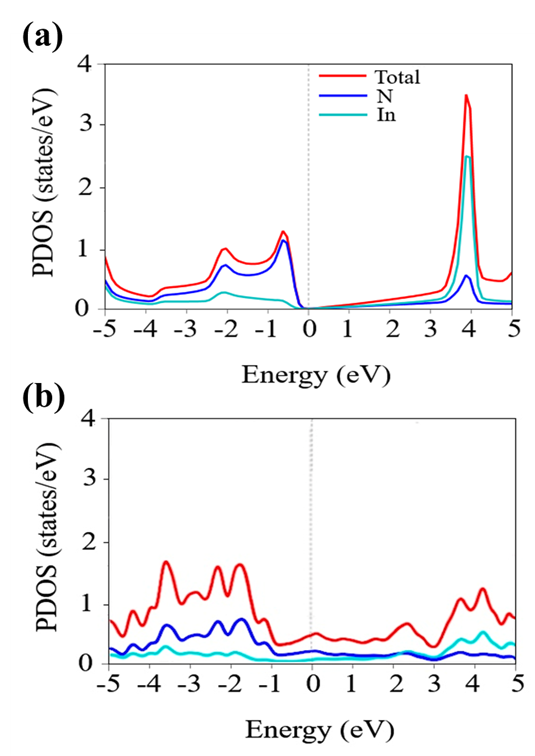


**Fig. S2.** Partial density of states (PODS) of the In and N atoms for the (a) monolayer InN and (b) Au-supported monolayer InN. The Fermi energy level is set at zero.

We have also demonstrated the atom projected density of states of the In and N atoms for the InN monolayer and Au/InN interface, as shown in **Fig. S2**. The Fermi level of the monolayer InN at the Au/InN interface is pinned near the conduction band, indicating that the interface is an n-type contact. This engenders an interlayer Schottky barrier with higher contact resistance between the Au/InN contact, leading to the restriction of the charge carrier transferring between the Au and InN interfaces. Our findings agree with earlier theoretical studies on metal/2D-materials contact^2–4^. Inductive charges are created when the electrode contacts the InN layer, according to the tribo-piezoelectricity mechanism described in Figure 5, and no charge transfers from the InN to the electrode. The charge transmission between the metal electrode and the InN layer will be hampered by the interface Schottky barrier. As a result, the metal contact has only a little impact on the InN bilayer's tribo-piezoelectricity. Furthermore, recent experiments on Au/TMD have also shown similar electronic properties that TMD layer is slightly influenced by the Au electrode^5,6^.

**References**

1. Cai, H., Guo, Y., Gao, H. & Guo, W. Tribo-piezoelectricity in Janus transition metal dichalcogenide bilayers: A first-principles study. *Nano Energy* **56**, 33–39 (2019).

2. Çakır, D., Sevik, C. & Peeters, F. M. Engineering electronic properties of metal–MoSe2 interfaces using self-assembled monolayers. *J. Mater. Chem. C* **2**, 9842–9849 (2014).

3. Popov, I., Seifert, G. & Tománek, D. Designing Electrical Contacts to MoS2 Monolayers: A Computational Study. *Phys. Rev. Lett.* **108**, 156802 (2012).

4. Su, J., Li, N., Zhang, Y., Feng, L. & Liu, Z. Role of vacancies in tuning the electronic properties of Au-MoS2 contact. *AIP Advances* **5**, 077182 (2015).

5. Brennan, C. J. *et al.* Out-of-Plane Electromechanical Response of Monolayer Molybdenum Disulfide Measured by Piezoresponse Force Microscopy. *Nano Lett.* **17**, 5464–5471 (2017).

6. Lee, J.-H. *et al.* Reliable Piezoelectricity in Bilayer WSe2 for Piezoelectric Nanogenerators. *Advanced Materials* **29**, 1606667 (2017).
